# Can Non-Economic Incentives Improve the Incentive Effect of Relocating Immigrants? Evidence from Wuwei City, Located at the Northern Foothills of the Qilian Mountains

**DOI:** 10.3390/ijerph192013039

**Published:** 2022-10-11

**Authors:** Ya Wang, Lihua Zhou

**Affiliations:** 1 Key Laboratory of Desert and Desertification, Northwest Institute of Eco-Environment and Resources, Chinese Academy of Sciences, Lanzhou 730000, China; 2 Institutes of Science and Development, Chinese Academy of Sciences, Beijing 100190, China; 3 School of Public Policy and Management, University of Chinese Academy of Sciences, Beijing 100049, China

**Keywords:** immigration, non-economic incentive factors, negative behavior, incentive effect, ANOVA, generalized linear regression, north foothills of Qilian Mountain

## Abstract

Towards the long-term sustainable development of the northern foothills of the Qilian Mountains, the design of migration motivation mechanisms is a key issue. Considering motivation perspectives and social exchange theories, a framework and measurement scale were constructed to analyze the influence of government incentives on Wuwei’s relocated immigrants. ANOVA and generalized linear regression models were employed to analyze the differences among regions and groups, along with the influence of various incentives. The results of the study indicate: (1) In Wuwei City, non-economic incentive factors were rated higher than economic incentives. The overall evaluation value for the negative behavior of immigrants was 0.369, and the incentive effect score was 0.633, with significant regional variation. (2) Based on cultural types, Wuwei City was found to have a high percentage of hierarchists, egalitarians and individualists, comprising 39.22%, 23.85% and 21.54%, respectively. Among cultural types, motivation factors and the incentive effect of the government made no difference except for the index of communication and opportunistic behavior. (3) Immigrants’ negative behavior was significantly affected by their cultural types. An economic incentive from the government increased the likelihood that immigrants would adopt negative behavior, whereas a participation incentive effectively eliminated those behaviors. (4) The government’s economic incentives appear significantly better at promoting the incentive effect than non-economic incentives, but in dispelling the negative behavior of immigrants, non-economic incentives have played a significantly greater role than economic incentives. Incentives associated with relocation provide a new objective basis for determining a policy scheme for the relocation of migrants in the Qilian Mountains, and departments can formulate corresponding incentive models and support policies accordingly as a consequence.

## 1. Introduction

Following the alleviation of rural poverty, a focus is being placed on the decline and revitalization of rural areas. As an indispensable and important object in rural reconstruction and overall revitalization, relocation of immigrants has affected a population of considerable size and typicality [1]. From 2001 to 2015, more than 1.2 × 10^7^ people were relocated for poverty alleviation, ecological migration and disaster prevention, according to statistics provided by the National Development and Reform Commission. In the 13th Five-Year Plan period, more than 9.6 × 10^6^ people were registered as poor in 1400 counties spread over 22 provinces (autonomous regions and municipalities). About 1 × 10^7^ people in southwest and northwest China will need to be relocated before 2050 to achieve ecological protection and common prosperity [2]. As of now, the majority of research on relocation migration focuses on the livelihood patterns and transition of migrants, livelihood risks and resilience, adaptation and integration (hometown attachment, identity, cultural reconstruction), reconstruction and sustainable development of livelihood spaces, follow-up industry development and long-term livelihood choices, poverty reduction effect and countermeasures, and a return to willingness and happiness [3,4,5,6,7,8]. The existing studies do not provide an in-depth analysis of migration capacity, vulnerability and resilience of resettlement sites’ social−ecological systems, space configuration and conflict [9], relocation experience, the public value and incentive mechanisms of migration projects [10]. At the key point of effective connection between poverty alleviation and rural revitalization, how can we realize “relocation, stability and prosperity” for immigrants, as well as prevent the risk of their return to poverty, back-migration and disorder in the process of livelihood transformation of migrants? It is urgent that we carry out case studies to summarize the relocation experience and lessons from it, and establish an incentive model.

As Guo points out, the current relocation of immigrants in China is not the result of market-driven behavior, but rather of a mixed social transaction led by the government [11]. Economic compensation and support for immigrants is determined by the state, and the grass-roots government, as an implementer of the policy, can only make adjustments within its framework [11]. Economic factors are limited in their ability to promote immigrants to “move out and stay stable,” which must be supplemented by non-economic incentives, such as honor, power, social status and trust. Observations from the perspective of social transaction theory indicate that there are many opportunistic behaviors and negative conflicts during the relocation process of immigrants. The government must complete the transformation of the incentive mode from economic to behavioral in order to reduce this behavior. Jia et al. argue that in order to effectively govern rural public affairs, it is imperative to fully consider the endogenous incentives of social preferences, while avoiding the crowding out effect caused by improper external incentives [12]. A key scientific challenge in the construction of long-term migration mechanisms is the ability to objectively evaluate the effectiveness of economic incentive mechanisms and non-economic incentive mechanisms, as well as the design of incentive mechanisms to improve migration initiative and stability. Currently, only a few scholars have considered the concept of incentives when discussing immigration. According to the incentive perspective and social transaction theory, Guo developed a structural equation model of non-economic incentive factors in large and medium hydropower project migration [11]. This study focuses on the incentive effect of commitment, trust, satisfaction and communication and their impact on negative behaviors of immigrants. It has two shortcomings: firstly, it does not analyze the influence of changes in key incentive factors on the incentive effect in multiple situations where different incentive factors are combined; secondly, it does not analyze the resolving and changing negative immigrant behavior in various combinations of intervention measures.

Wuwei City, which has attracted large number of immigrants to the northern foothills of the Qilian Mountains, shows significant project benefits and prominent practical problems. Our study selected this area’s “ecological migration from hills into the flat area” and “poverty alleviation [and] relocation [of] immigrants during the 13th Five-Year Plan period” as its object. It examined the non-economic incentives, immigrants’ negative behavior and incentive effect across regional and cultural groups using ANOVA. The generalized linear regression model was employed to clarify the influencing mechanisms and role changes of each incentive factor, in order to determine whether non-economic incentive factors can effectively improve the incentive effect of immigrants and whether there is a difference between immigrants’ negative behavior and incentive effects when different incentives are combined. The goal was to seek the best mix of incentives for the local area and to provide case support for the development of appropriate motivation policies for subsequent migration to the Qilian Mountains.

## 2. Material and Methods

### 2.1. Overview of the Study Area

**Figure 1:**
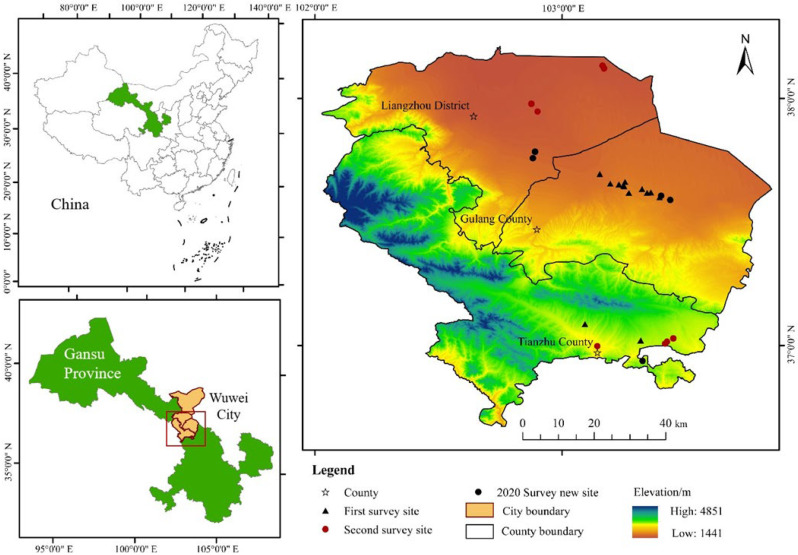
Administrative division map and survey points of Wuwei City.

The Qilian Mountains are located at the intersection of the Loess Plateau, the Qinghai-Tibet Plateau and the Mongolian-Xinjiang Plateau. They are divided into north and south foothills according to the boundary between Gansu and Qinghai Province. The northern foothills mainly cover Wuwei, Zhangye, Jinchang, Jiuquan and Jiayuguan in the Hexi Corridor in Gansu Province. With the improvement of ecological management and construction of national parks in the Qilian Mountains, this area has become an area of national ecological sustainability and green development. As the most typical topographic transitional zone and ecologically fragile part of this area, Wuwei City (101°49′–104°16′ E, 36°29′–39°27′ N) serves two functions: protecting water from the south and preventing desertification from the north (Tengger Desert to the north and the Qilian Mountains to the south) (Figure 1). This area is dry, rainy and windy, with a total area of 3.32 × 10^4^ km^2^, of which 65.5% is desert. Approximately 33.37 × 10^2^ km^2^ of the city area is devoted to the Qilian Mountain National Park. As of 2020, Wuwei City had jurisdiction over one district (Liangzhou) and three counties (Tianzhu, Gulang and Minqin), with a total population of 195.23 × 10^4^, the agricultural population accounting for 52.99%. Initially, the city formed a trend of multi-business development of “agriculture +” by developing “three characteristic industrial belts” along mountains, rivers and sand, and “eight industries” of cattle, sheep, chicken, vegetables, fruit, edible mushroom, potato and Chinese herbal medicine.

### 2.2. Overview of the Relocation Project in Wuwei City

For almost 30 years, Wuwei City has carried out various relocation projects since Minqin County “developed the South Lake and migration with water” in 1991. The most typical examples include the ecological migration project of farmers and herders from high-altitude areas to flat areas, which was launched in 2011, and the poverty alleviation relocation project, which was launched in 2016. For the former, farmers and herds living above 2800 m and in “four zones” (reservoir area, mining area, subsidence area, ecological core area) were primarily relocated to flats or towns via a long-distance and centralized resettlement method. The “poverty alleviation relocation project” mainly relocated the residents (including the registration of poor households and synchronous relocation of non-poor households) in the Qilian Mountains, whose ecology is fragile and vulnerable to geological disasters, to the resettlement sites of Huanghuatan, Nanyang Mountain and Dengmaying Lake. A total of 17.02 × 10^4^ people have been relocated from 4.42 × 10^4^ households since the 12th Five-Year Plan began, and 93 resettlement areas have been built. As a result of the topography, landform, and geographical resources (water and transportation) of mountainous areas in the south, narrow oases in the middle and flat deserts in the north, migrants in this city are primarily settled on the edge of typical sand areas or desert areas. The resettlement sites suffer from a seasonal shortage of water, a large outflow of population, aging villages, and a deficit of development opportunities and supporting industries. How to implement migration relocation well has become an urgent problem to be solved in the process of promoting the green and high-quality development of Wuwei City.

### 2.3. Data Sources and Sample Analysis

A three-stage survey was conducted: first, household surveys of relocated immigrants were conducted in 13 typical resettlement villages (Huangcaochuan, Lixiang, Deji in Tianzhu County; oasis towns, Aimin, Fukang, Huimin, Yuanmeng, Weimin, Yangguang, Ganen, Kangle and Fumin in Gulang County), and face-to-face interviews with village cadres in Songshan town (Tianzhu County), Heisongyi, Shibalibao and Huanghuatan (Gulang County). The focus of the first survey was to understand the impact of relocation on the livelihood and development of migrants, the relocation willingness and policy needs of migrants in the relocation area, government support measures and industrial development orientation. Second, 55 immigrants were randomly selected for the “Non-Economic Incentive Questionnaire” and “Migrant Relocation and Livelihood Questionnaire” pre-survey. They came from the new villages of Huajitang, Xiujie, Zijinghuayuan (Tianzhu County) and Xinzhuang, Changcheng, Hongshuihe, Ronghua and Fuqiang (Liangzhou District). We then conducted face-to-face interviews with the village cadres in Jiuduntan ecological construction headquarters, Changcheng town and Hongshuihe village (Liangzhou District). Afterward, the questionnaire was modified, the survey scheme was improved, and questionnaire investigators were trained. In Tianzhu, Gulang and Liangzhou, 18 typical migrant villages were surveyed, and 131 valid questionnaires on “non-economic incentives” were returned, accounting for 33.59%, 45.80% and 20.61% in each location.

**Figure 2:**
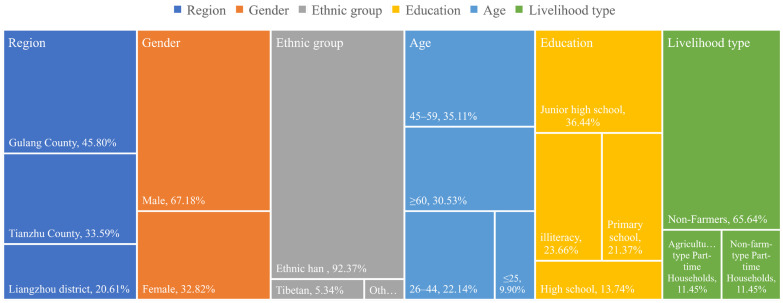
Characteristics of surveyed respondents.

A total of 67.18% of respondents were males, 92.37% were Han nationals, 65.64% were over the age of 45, and 17.56% had a high school education or higher (Figure 2). Respondents had an average household size of five, a population burden ratio of 0.464, and a livelihood diversification index of only 1.74. Out-migration for work was the major source of income. The proportion of non-farm households accounted for 65.64% of the total sample, with an average non-farm income of CNY 29,030 per household. Liangzhou District, Gulang County and Tianzhu County allocated land according to per capita standards of 0.133 hm^2^, 0.117 hm^2^ and 0.100 hm^2^. As a consequence of the lack of irrigation water and the predominantly sandy soil, approximately 32.82% of immigrants transferred the newly acquired land to local planting enterprises. The average household transfer income was CNY 1881 per year. Under the influence of capital allocation, economic shock and livelihood transformation, 67.44% of the respondents were threatened by multiple risks such as economic, policy, welfare, health and security [6]. There was a risk of returning to poverty and disorder at the family and community scale due to disconnection and system mismatching. Yet 20.1% of immigrants believed that their living standards would definitely improve in the next five years with the implementation of a series of supporting policies, including poverty alleviation, targeted poverty alleviation and rural revitalization, and only 13.6% believed that they would decrease in the next five years. The future livelihood strategy of expanding breeding scale was preferred by 39.52% of the respondents.

### 2.4. Methods and Models

#### 2.4.1. Division of Cultural Types of Immigrants

**Figure 3:**
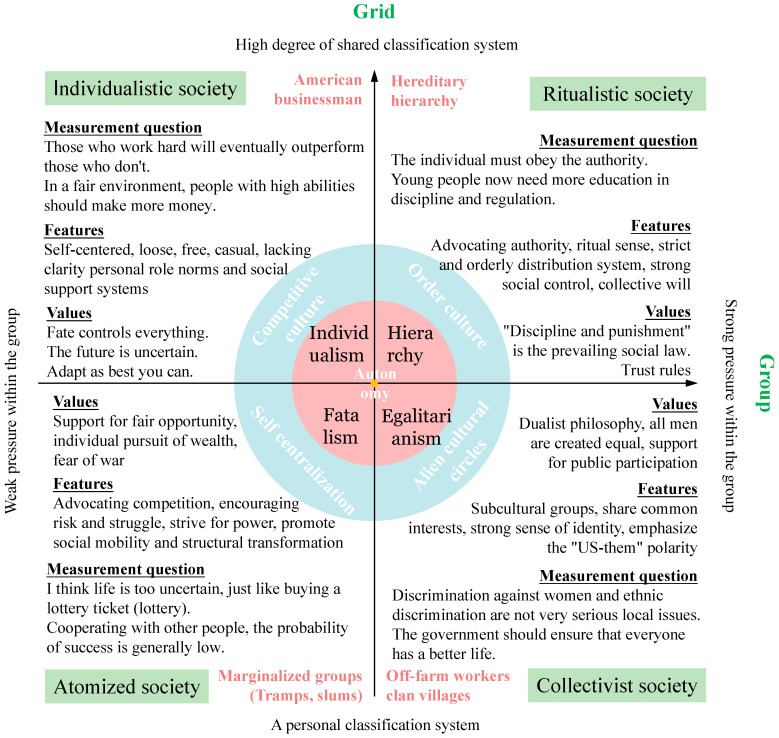
Classification matrix and determination of cultural types. This figure refers to the reference [13,15,16,17,18,20,21] in its drawing. The theoretical basis for this figure lies in Mary Douglas’s culture theory of the grid−group. The measurement question of each culture type is set up with reference to the literature [20,21].

The grid−group culture theory, based on Emile Durkheim’s structuralism theory and George Boole and Garrett Birkhoff’s “grid theory”, provides a powerful explanation for complex social phenomena [13]. It is widely used to analyze cultural types and international relations, causal and structural links between social groups and risk selection, the influence of organizational culture on new product development performance, corporate cultures, policy analysis and public management research. Hood uses “grid−groups” to classify the basic organizational types of public administration models in various countries since ancient times [14]. He discusses whether a good institution is primarily governed by rules, laws, or the judgment of wise men from the perspective of the “grid”. Furthermore, he discusses how to coordinate and build a governance network among professionals, private businesses and ordinary people in a good system from the perspective of the “group”. Zhang believes that the current network class has the characteristics of a “weak grid” and “strong group” [13]. Administrative management is weakened, conflicts are focused and cyberspace governance is difficult in this scenario. These problems can be solved by changing the pattern of “group” and “grid”, using class interaction, network opinion leaders, polycentric governance and moderately strengthening management. *Natural Symbols*, a book by Mary Douglas published in 1970, was the first to propose the “grid−group” theory. She divided the social types into ritualistic, collectivist, individualistic and atomized society through the power direction (grid refers to the degree of consistency within the public classification system, i.e., their structural position) and status direction (group, that is, an individual’s choice of action is influenced by the pressure within their group), and corresponding sequences in turn order culture (hierarchy), alien cultural circles (egalitarianism), competitive culture (individualism), self-centralization (fatalism) and autonomy (the origin) of five cultural types [15,16,17,18,19], and the complex groups, simple groups, individualist, isolationist and reclusive groups (Figure 3).

Yin et al. [20] and Zhong et al. [21] used Dake’s questionnaire design idea of cultural bias and risk structure to design 28 corresponding measurement questions regarding opinion preferences across various cultures. The cultural type of the respondents can be determined by their level of agreement with each view (score 1 to 5 on the degree of agreement). The determination methods are as follows: first, select the measurement questions (Figure 3) and calculate the average recognition of each sample for each cultural type and the average value of the total sample. Second, if one type of index is higher than the overall average, and the other types are lower than the overall average, the higher is judged as the cultural type. Third, if multiple indicators are greater than the average value, the indicator type with the largest difference will be used as the judgment type. Fourth, a type is autonomous if the values of each type are lower than the average value. In summary, the grid−group culture theory provides a new perspective on government motivation, which can be used to study differences among groups, clarify problems and their crux and aid in developing targeted adjustment strategies.

#### 2.4.2. Framework and Indicators

As an important concept in management science, motivation refers to a series of activities in which the motivation subject’s overall goal and interest are the starting point and the internal unmet needs of the object are the basis to mobilize and strengthen the object to achieve the overall goal and interests of the subject [22,23]. The motivation theory concept originated in the Renaissance in the late 18th century. It has undergone a historical evolution from that of a single monetary stimulus to meet various needs, to the generalization of incentive conditions and the clarification of incentive factors, and from the basic research of incentive to the exploration of incentive process [23,24,25]. It can be divided into economic motivation theory and management motivation theory. The economic motivation theory is based on the assumption of a rational economic individual and utility maximization, and has formed the contract theory system, including transaction costs, incomplete contracts and enterprise ownership allocation. It examines the interaction between incentive subject and object as well as the transaction cost between information and agent. Based on the different research content and behavior relationships between the subject and object of incentive, motivation theories of management can be divided into four categories: content, behavior modification, process and comprehensive incentive models [23,26]. Among the applications of the above theories are enterprise supervision, functional benefit evaluation of government departments and project performance management.

**Figure 4:**
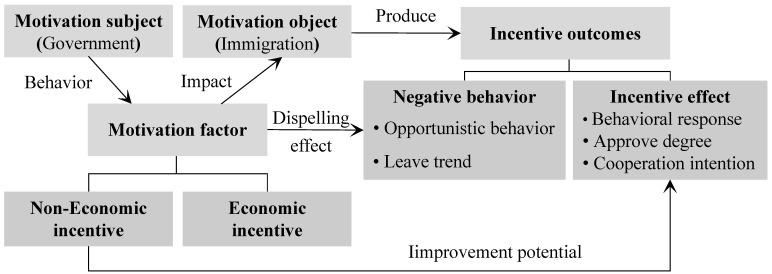
Framework of immigration incentive mechanism model.

In contrast to economic incentives, which use material means (salaries, bonuses, property rights, welfare) to meet low-level needs, non-economic incentive is an implicit incentive. It pays more attention to constructing the mechanism of trust, coordination, satisfaction and reciprocity between the subject and object of an incentive by means of communication, commitment, reward, encouragement, punishment, restraint, publicity, recognition, praise, administration and law, so as to avoid moral hazard [27,28,29]. Examples include reputation incentive mechanisms and entrepreneurship. From the perspective of an interactive game between the subject and object of an incentive, Huang [23] and Guo [11] examined the influence of incentive factors on the incentive effect. Based on the content type motivation theory and social exchange theory, they decomposed the motivation factors into commitment, trust, communication and satisfaction, and decomposed the incentive effect into negative behaviors (opportunistic behavior, tendency to leave, conflict and decision uncertainty) and positive responses (response, recognition, cooperation, satisfaction) of the incentive object [11,23,30]. This study draws on the framework and measurement problem of two scholars, combined with the project objective−implementation process−reconstruction results of the migration project and the “phenomenon−recognition−perception−behavior response−causality” analysis paradigm to construct the three-layers framework (Figure 4) which covers subject incentive factors, the forward and reverse effect evaluation of the incentive object and their causal relationship. In this framework, motivation, non-economic incentives and incentive outcomes are the core concepts. The government and the immigrant are the subjects and objects of incentives, respectively. As the core of the framework, motivation factors are decomposed into non-economic factors and economic factors, among which non-economic incentive factors include commitment, trust and communication. An incentive outcome is composed of the negative behavior and the positive effects of the motivation object. Immigrants’ opportunistic behavior and tendency to leave are used to reflect their negative behavior choices, the level of incentive effect is measured by immigrants’ response to migration, recognition and willingness to cooperate. The logical premise of this framework is that the government, as the motivation subject, will adopt some incentive means to encourage the motivation object (immigrant) to take positive actions that result in certain incentive outcomes. As a second logical thread, government incentives may eliminate some of the negative behavior of immigrants. The third layer logic proposes that non-economic incentive factors may be effective in improving incentive effects. As a result of the framework, the checklist of all elements, the logical correlations and context structure of each level of analysis have been clarified. It followed the logic that economic and non-economic incentive measures taken by the government would stimulate the initiative of immigrants themselves, reduce their negative behaviors and produce incentive effects.

The degree of economic incentive of the government is embodied by the degree of agreement with the statement “the compensation and support conditions offered by the local government are very attractive to us”. In terms of commitment, it refers to the effort and guarantee made by the government in order to maintain the relationship with immigrants. Government commitment is assessed by participation motivation, respect incentives and vision incentive evaluations of immigrants to the government. As a sign of the reciprocity and success relationship, trust is the immigrants’ confidence in the goodwill and reliability of the government, which is reflected in the immigrants’ perception of the sense of gain, security and trust in the process of relocation. It can be measured by the evaluation of the immigrants’ trust motivation, development incentive and spiritual motivation to the government. Timely, comprehensive and in place policy publicity, formal or informal communication and consultation with immigrants, are effective measures for communication and incentivization, which can effectively reduce conflicts and contradictions caused by information errors. In accordance with the content of social transaction theory, negative behavior of an motivation object includes two aspects: opportunistic behavior and conflict. Huang [23] expanded the tendency to leave and the uncertainty of decision-making in his incentive study of channel members. It has been very common for immigrants to engage in opportunistic behavior during the relocation process of the Three Gorges Reservoir area and during the migration process of the project. This is a type of fraudulent profit-seeking behavior that violates the terms of a formal or informal contract, which can be measured by its profit-seeking behavior and breach of contract. With a high cost of adaptation to the new life and difficulty in integrating into the existing life and cultural atmosphere in the resettlement place, such immigrants will have a high likelihood of terminating the cooperative relationship by moving out again. Their intention to migrate directly predicts their tendency to leave. From immigration response (characterized as “move out”), effect recognition and cooperation intention (characterized as “live in stability”) to feedback the incentive effect of incentive object. The recongution can be subdivided into the paradise recognition and the approve of government work, and the cooperation can be reflected from the compromise and advice feedback made by immigrants.

In Table 1, 17 indicators were tested for reliability and validity using SPSS 22.0 (IBM Corporation, Armonk, NY, USA). A Cronbach’s coefficient of 0.718 was found for the total scale, indicating good reliability. The KMO value was 0.730, and the *p* value of the Bartlett spherical test indicated good validity at the 1% level. Range standardization and comprehensive evaluation were used to calculate the comprehensive scores of incentive factors, negative behavior and incentive effect. In terms of the average value of each index, immigrants were most highly rated on the government’s propaganda incentive (3.885) and global awareness of the incentives’ effect (3.878), followed by the economic incentives (3.641) and migration responses (3.763). As far as negative behaviors of immigrants were concerned, profit seeking and defaulting were less prevalent and the score was low. Due to the fact that the combination weighting method in game theory is a biased combination method for finding the equilibrium point or the optimal combination by Nash equilibrium, it presents the problem that the weight coefficients do not equal 1, nor do the weights equal 1. Thus, the combined weight in this study was calculated using the simple linear weighting method. According to the average perspective, the weight coefficients of entropy weight, CRITI and coefficient of variation were assigned 1/3. The combined weight results show that, among the weight of government incentives, the trust motivation (0.338) > commitment incentive (0.328) > communication incentive (0.214) > economic incentive (0.120). In terms of evaluation scores for incentive factors, the trust motivation was the largest (0.133), while the development incentive was the smallest (0.800). The weight of opportunistic behavior (0.741) was significantly greater than the weight of emigration tendency (0.259). In terms of incentive effects, the relocation response weight was highest (0.240), followed by recognition of the “paradise” (0.199) and feedback (0.196). While overall consciousness of immigrants had the highest evaluation value, the index weight (0.171) was low. ANOVA was used to analyze the differences between regions and cultural types of immigrants.

#### 2.4.3. Model and Hypothesis

It is necessary to study the influence of government incentive factors on the negative behavior of and incentives for immigrants, as it enables identification of development obstacles and enhancement of the potential of immigrants’ relocation and resettlement processes, as well as developing a plan for future migration to the northern foothills of the Qilian Mountains. Accordingly, assuming that the government’s motivation factors can facilitate immigration initiative towards reducing negative behavior and thereby produce corresponding incentive effects, then:

**Hypothesis** **1** **(H1).** *Immigrants’ perception of various non-economic incentives is negatively correlated with their negative behaviors.*

**Hypothesis** **2** **(H2).** *Immigrants’ perception of various non-economic incentives is positively correlated with their incentive effects.*

**Hypothesis** **3** **(H3).** *The dispelling effect of economic incentives on negative behaviors of immigrants is significantly lower than that of non-economic incentives.*

**Hypothesis** **4** **(H4).** *The promoting effect of economic incentives on the incentive effect of immigrants is significantly lower than that of non-economic incentives.*

**Hypothesis** **5** **(H5).** *Ethnic attributes, cultural types, education level and location significantly affect the negative behaviors of immigrants.*

**Hypothesis** **6** **(H6).** *Ethnic attributes, cultural type, education level and location significantly affect the incentive effect of immigrants.*

In order to answer the above hypotheses, this study used a generalized linear regression model to successively set the negative behavior of immigrants, incentive effect and its sub-score as the dependent variable *Y_i_*, and selected all motivation factors of the government as explanatory variables. Considering the influence of geographical and spatial location, education level, cultural type and ethnic factors of immigrants, Tianzhu County, education level of primary school or below, fatalism and Han nationality were taken as control variables. The model was constructed as:

(1) $$Y_{i}=\beta_{0}+\beta_{1}X_{1}+\beta_{2}X_{2}+\cdots +\beta_{n}X_{n}+\varepsilon$$

where $Y_{i}$ is the score of negative behavior, incentive effect and its component, $\beta_{0}$ is a constant, $\beta_{1},\beta_{2},\cdots ,\beta_{n}$ is the regression coefficient, $X_{1},X_{2},\cdots ,X_{n}$ represents each explanatory variable.

## 3. Results

### 3.1. Difference Analysis of Incentive Factors and Their Influences

#### 3.1.1. Space Differences in Motivating Factors, Negative Behaviors and Incentive Effects

**Figure 5:**
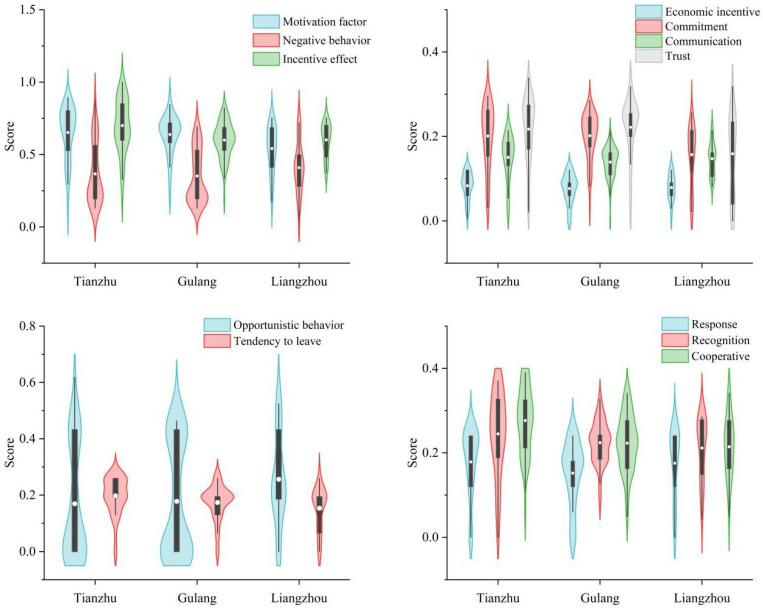
The scores of motivation factor, negative behavior and incentive effect for migrants in different regions.

Wuwei immigrants were rated at 0.079 on the evaluation of government economic incentives (Figure 5), with Tianzhu County having the highest score (0.083), followed by Liangzhou District (0.079) and Gulang County (0.076). There was no significant difference between regions (Levene statistic was 0.286, and the variance between groups was homothetic, and *p* = 0.598). The overall evaluation value of immigrants for the government’s non-economic incentive factors was 0.623, of which the trust motivation dimension scored the highest (0.207), followed by commitment (0.192) and communication (0.144). Significant differences were observed between regions (Levene statistic 2.750, homogeneity of variance between groups, *p* = 0.013). The score of Tianzhu County (0.651) was higher than that of Gulang County (0.638) and Liangzhou District (0.541). According to the post hoc multiple test results of LSD and S-N-K, Liangzhou District government scored significantly differently from Tianzhu County and Gulang County for non-economic incentive factors (*p* values were 0.006 and 0.01, respectively). In terms of government commitment incentive, the immigrants from Gulang County were rated the highest (0.220), while those from Liangzhou District were rated the lowest (0.157). The difference between Liangzhou District and Tianzhu County, and Gulang County (Levene statistic was 3.240, *p* = 0.008) was significant. Tianzhu County ranked highest in terms of communication between the government and immigrants (0.150), while Gulang County ranked lowest (0.139). Statistically, there was no difference between regions (Levene statistic was 2.759, and variance between the groups was identical, but *p* = 0.381). Immigrants from Gulang County had the highest trust in the local government (0.221), whereas immigrants from Liangzhou District had the lowest (0.159). Liangzhou District and Tianzhu County, and Gulang County had significant differences (Levene statistic 3.432, *p* = 0.002). In conclusion, there were significant regional differences in the evaluation of non-economic incentive factors of immigrants to the government, among which there were significant differences between groups in commitment and trust. Liangzhou District had the lowest score among the three places, which was significantly different to that of Tianzhu County and Gulang County.

The overall evaluation value of negative behaviors of immigrants in Wuwei City was 0.369, and the scores of opportunistic behaviors and tendency to leave were 0.191 and 0.177 (Figure 5), with no difference among regions (Levene statistic was 3.088, *p* = 0.430). The score of Liangzhou District (0.410) was higher than that of Tianzhu County (0.366) and Gulang County (0.352). The Liangzhou District immigrants scored highest (0.256) in terms of opportunistic behavior, whereas Tianzhu County immigrants scored lowest (0.169), with no significant differences between the two regions. Wang Ya et al. found that approximately 96.90% of immigrants in Wuwei City experienced livelihood risks after relocation, with immigrants in Liangzhou District experiencing the highest levels of livelihood risks: 29.63% of immigrants experienced four or more risks, with the highest risk combination being “government subsidies for losing grassland and farmland + returning to poverty” (29.53%) [6]. Of the surveyed immigrants, 4.30% believed that there were cases of lying about the intention and quantity of farming to obtain government subsidies. Tianzhu County had the highest tendency to return to the place of origin (0.197), while Liangzhou District had the lowest tendency (0.153). A significant difference was found between Tianzhu County and Liangzhou District, Gulang County (Levene statistic was 1.661, with the same variance between groups, *p* = 0.026). Typical of grassland pastoral areas, Tianzhu County has 54.90% natural grassland; most settlers retained pastures for grazing. A majority of migrants in this county moved from mountainous to plain areas and changed their livelihood mode from traditional grazing and rainfed farming to refined house feeding and irrigation farming. The adaptation cost and livelihood risk were the highest among the three places. Moreover, the new sub-farmland quality is poor and the area is small, lacking irrigation water, so it is not feasible to plant anything. The only alternative is to go out to work. About 75.00% of immigrants were not satisfied with income, and their desire to move back was the strongest. For those over the age of 60, the only option is to return to their original places and continue planting and breeding to support their families.

Despite the influence of economic incentives and non-economic factors of the Wuwei City government, the incentive effect score was 0.633, with the highest score of immigrant cooperation (0.239), followed by effect recognition (0.228) and behavioral response (0.166). A significant difference was observed between Tianzhu County and other regions (Levene statistic was 4.818, *p* = 0.001). Tianzhu County demonstrated the most prominent incentive effect (0.700). In terms of behavior response and recognition, there was no difference among regions (Levene statistic was 0.237 and 9.383, *p* values were 0.183 and 0.153). The highest scores were in Tianzhu County (0.179 and 0.245), the lowest scores of response were in Gulang County (0.152), and the lowest scores of recognition in Liangzhou District (0.211). However, there were significant regional differences in the willingness of immigrants to cooperate with the government (Levene statistic was 0.591, with the same variance between groups and *p* = 0.000), and Tianzhu County showed the strongest willingness among immigrants to cooperate with the government. In conclusion, there were significant regional differences in the incentive effects of non-economic incentive factors from the perspective of immigrants, and Tianzhu County had the greatest difference in cooperation willingness.

#### 3.1.2. Cultural Types Differences in Motivating Factors, Negative Behaviors and Incentive Effects

**Figure 6:**
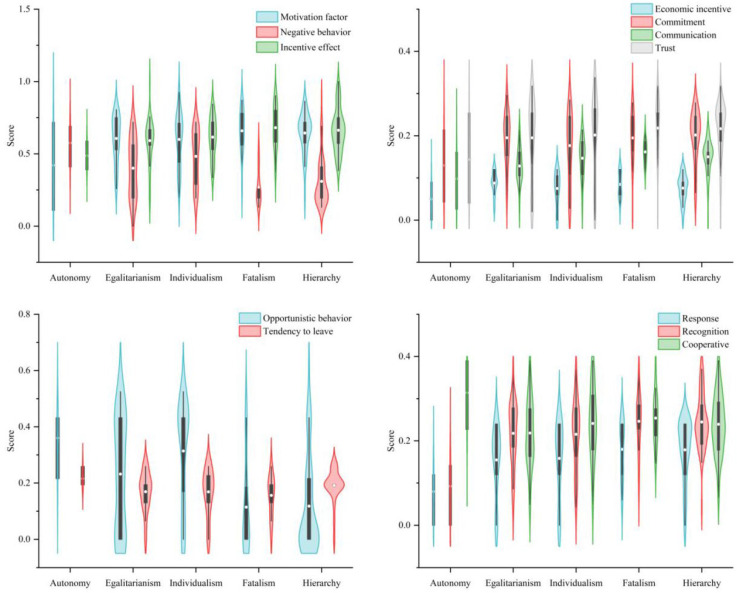
The scores of motivation factor, negative behavior and incentive effect for migrants of different culture types.

In Wuwei City, fatalists, individualists, egalitarians, hierarchists and autonomists made up 13.08%, 21.54%, 23.85%, 39.22% and 2.31% of the total sample, respectively. In terms of economic incentive, all types of respondents were evaluated similarly (Levene statistic = 2.498, *p* = 0.203). The score for egalitarians was 0.088 (Figure 6), followed by fatalists (0.085), hierarchists (0.076) and individualists (0.075), while the autonomists scored the lowest (0.050). Egalitarians belonging to the “high group and low group” have clear group boundaries and close interpersonal relations, but no rigid group norms. According to them, equality is a core value, public participation is important, and nature must be protected by reducing demand on it. Approximately 77.42% of egalitarians believed that the compensation and support conditions provided by the local government were very attractive to immigrants, while 51.61% of immigrants agreed with it, while 25.81% strongly agreed. According to the evaluation of non-economic incentive, immigrants’ commitment incentive and trust motivation towards the government were similar, and there was no inter-group difference in cultural types. Other findings were: 51.54% of immigrants believed that local government placed a high value on cooperating with immigrants, listened to their comments and made adjustments to meet their needs; 64.62% of immigrants agreed that the local government considered immigrants’ problems when making decisions; 66.92% of immigrants believed that their local government cared about their safety, especially when it involved their interests as immigrants. Fatalists believe the future is uncertain, and they can only adapt to changes in the outside world by following strict norms. Therefore, fatalists placed the greatest trust in government (0.218), followed by hierarchists (0.216) and autonomists (0.098). In terms of commitment incentive, fatalists and egalitarians scored highest (both 0.195), while autonomists scored lowest (0.130). However, the communication incentive between the government and immigrants differed significantly between groups (Levene statistic was 1.464, homogeneity of variance between groups, *p* = 0.018). Among fatalists and hierarchists, communication incentive rated highest (0.162 and 0.150), while autonomists rated it lowest (0.098). The fatalist in the “high grid and low group” differs markedly from the egalitarian and the autonomist, as does the hierarchist, who values order and authority. As a result, there was no difference in the cultural types of government incentive factors between groups, except for the communication incentive.

A significant difference was found in the negative behavior evaluation of different cultural types (Levene statistic of 2.366, homogeneity of variance between groups, *p* = 0.000). The scores of the autonomists were the highest (0.576). Fatalism had the lowest score (0.271) and was significantly different from that among individualists, egalitarians, and autonomists (*p* ≤ 0.015), and hierarchists were also significantly different from the above three (*p* ≤ 0.024). Among immigrants of different cultural types, opportunistic behaviors differed significantly (Levene statistic = 1.064, homogeneity of variance between groups, *p* = 0.000), and these differences were mainly between fatalists, hierarchists and individualists, egalitarians and autonomists. Following relocation, immigrants generally faced a dilemma of “double lack” of opportunities for development and support industries for the transformation of their livelihoods. About 41.41% of respondents were not satisfied with the current family income, while 50.39% just achieved a balance between income and expenditure. With too much expenditure, 19.69% of respondents believed it was difficult to live and tended to return to poverty (29.01%); 20.61% of immigrants believed that adapting to the new production and lifestyle was very expensive [6]. Among respondents, 15.69% of hierarchical, 14.29% of individualists and 11.76% of fatalists desired to move to another state or return to their hometown. However, 19.35% of egalitarians would like to move again or return to their original location to ease the livelihood pressure. Even when immigrants from different cultural groups faced the same risks and difficulties, their tendency to leave did not differ significantly.

Among immigrants of different cultural backgrounds, there was no significant difference in incentive effects (Levene statistic was 0.283, homogeneity of variance between groups, *p* = 0.057). In terms of sub-effects, behavioral response and willingness to cooperate did not differ significantly between groups (Levene statistic was 0.674 and 0.553, with homogeneity of variance between groups, *p* values of 0.176 and 0.214, respectively). Among the five cultural types, fatalism had the most significant incentive effect (0.680), resulting in the highest scores in behavioral response, effect recognition and cooperation intentions. Autonomism, however, had the worst incentive effect (0.487), and the lowest behavioral response and effect recognition scores. Immigrants from Wuwei City were highly recognized for local government (0.228) and 19.23% of respondents did not wish to change the immigration cadres. In addition to 36.92% of immigrants being willing to praise the work of the local government to the media or friends, 33.08% of immigrants agreed “the new village is a beautiful paradise in our mind”. However, among immigrants with different cultural types, there were significant group differences in the recognition of government work (Levene statistic = 0.875, homogeneity of variance between groups and *p* = 0.004). The recognition of autonomists was significantly different from that of other cultural types (*p* ≤ 0.005).

### 3.2. The Influence of Motivation Factors on Negative Behavior and Incentive Effect of Immigrants

#### 3.2.1. The Dispelling Effect of Motivation Factors on Negative Behaviors

According to the multiple linear regression of negative behavior, the tolerance of each equation < 1, the variance inflation factor VIF < 3.590, and the Durbin−Watson values of models 1–8 were all close to 2 (Table 2). The residuals were independent and did not show any collinearity. In all cases, except for models 1 and 8, the explanatory power of the 13 variables included in the regression models exceeded 21.60% (adjusted R^2^ > 0.216 and *p* = 0.000), demonstrating a high degree of good fit.

In Model 1, the individual’s ethnic, educational, region and government’s economic incentives have no significant impact on the negative behavior of immigrants (Hypothesis 5 is not valid). In eight models (Table 2), immigrant culture type had a significantly positive influence on negative behavior at the 1% level. Individualists, egalitarians and autonomists were more likely to exhibit negative behaviors than fatalists. The above three types of people are all members of “low grid” groups, which means there are no strict norms within the group, members do not adhere to the norms, and individual behavior is free. Therefore, there is a high probability of negative behaviors. When joined with the commitment incentive (Model 2), the economic incentives of the government began to significantly and positively affect the negative behavior (*p* ≤ 0.05). Accordingly, after adjusting for collinearity between economic incentives and other variables, the negative behaviors of immigrants increase by 0.03 units for every 1 unit increase in government economic incentives (Hypothesis 3 is not valid). In contrast, when the government increases its participation motivation by one unit, immigrants’ negative behavior decreases by 0.069 units (Hypothesis 1 is valid). When the communication motivation is added (Model 3), the influence coefficient of economic incentives is significantly increased, and the government’s commitment incentive has a weaker dispelling effect on immigrants’ negative behaviors. Government policy publicity and communication did not effectively reduce immigrants’ negative behaviors (*p* ≥ 0.149). Introducing the trust motivation, a non-economic incentive factor, does not significantly affect the negative behavior, as well as the influence coefficient and significance of economic incentive and commitment incentive (Model 4). When the economic incentives are excluded and only the non-economic incentive factors are included, the results indicate that, except for the participation incentives significantly eliminating the negative behaviors (*p* < 0.01), other commitment, trust and communication factors do not significantly affect the generation of negative behaviors (Model 5). Trust and communication do not play a significant role in the totality of non-economic incentive factors. The participation motivation significantly reduces the negative behaviors at the level of 1%, while the economic incentive will promote the occurrence of negative behaviors. More specifically, the more the government takes the opinions of immigrants into account and attaches importance to their views, the less likely immigrants are to engage in opportunistic behaviors such as lying, cheating and violating rules during relocation (Model 7). More likely than not, the tendency of migrants to leave is due to their livelihood difficulties after relocation and the impact of multiple risks such as economic, policy and welfare, rather than the government’s economic and non-economic incentives in the process of relocation and rural reconstruction. Its explanatory power is only 6.3% and the *p*-value of Model 8 is not significant.

#### 3.2.2. The Potential Improvement of Motivation Factors in the Incentive Effect of Immigrants

In the multiple linear regression of the incentive effect of immigrants, the tolerance of each equation is all < 1, the variance inflation factor VIF is all < 3.539, the Durbin−Watson value of models 1–9 is about 2, there is no collinearity problem and the residuals are independent. Except for in Model 1 and 8 (Table 3), the explanatory power of the 13 variables included in the regression model for the incentive effect of immigration reached more than 22.9%, with a high goodness of fit. Ethnic, education (except cooperation Model 9) and cultural type (except Model 8) of immigrants had no significant influence on the output of incentive effect. At the 1% level, the output of the incentive effect of immigrants in Gulang County was significantly lower than that in Tianzhu County.

In Model 1, economic incentives have a significant positive impact on the output of the incentive effect at the level of 1%. For every unit increase in the government’s economic incentives, the incentive effect of immigrants will increase by 0.046 units after adjusting for collinearity between economic incentives and other variables. Gulang County and Liangzhou District had a lower economic incentive effect than Tianzhu County. In Model 2, the commitment incentive decreases the influence coefficient of economic incentives, which means that immigrants’ incentive effect can only be increased by 0.036 units for every unit increase in economic incentives. As a consequence of the government’s efforts toward the long-term development of immigrants after relocation (prospect incentive), immigrants will be more confident in their future prospects, stimulate endogenous motivation in the poor, form an interactive integration pattern for the promotion of income by industry and significantly increase the incentive effect output of immigrants (*p* = 0.04). The positive impact of economic incentives is enhanced when the communication variable is introduced. The incentive effect is increased by 0.027 units for every 1 unit increase in the government’s publicity incentive. Considering only the commitment and communication incentive, excluding the economic incentive factor, the impact of other commitment and communication factors is not significant (Model 4), with the exception of the prospect incentive, which will enhance the incentive effect of immigrants (*p* = 0.038). Immigrant incentives’ effect increases by 0.045 units at the 5% level when the government’s development incentive increases by 1 unit. To sum up, the commitment and communication between non-economic incentive factors can only be effectively promoted by the stimulus of economic incentives, and only the trust incentive based on commitment and communication can effectively contribute to the effect of immigration incentives. The government’s non-economic incentive factors play a significantly greater role in dispelling the negative behavior than its economic incentives, but in terms of promoting the incentive effect, economic incentive (influence coefficient and significance) plays a much more significant role than non-economic incentive factors (Hypothesis 4 is not valid).

According to the sub-items of incentive effects, economic incentives have a significant positive influence on immigrants’ relocation behavior. By increasing the index by one level, immigrants’ behavior response increases by 0.024 units. Among the non-economic incentive factors, only the spiritual incentive in the trust factor was significant at the 5% level, and its effect was only 0.833 times that of the economic incentive. In terms of effect recognition and cooperation intention, the effect of economic incentive is no longer significant, while the commitment incentive has a significant effect at the 5% level. Government vision incentives are increased by one level when the effect of recognition of relocation increases by 0.014 units. Immigrants and the government are more likely to cooperate when the government respects and complies with their will and needs (0.025). Gulang County and Liangzhou District showed significantly lower willingness to cooperate with government than Tianzhu County.

## 4. Conclusions and Discussion

Relocation immigration is an indispensable and important object in rural revitalization. Based on the incentive perspective and social transaction theory, this paper constructs an incentive mechanism analysis framework for the relocated immigrants in Wuwei City, and systematically analyze the regional and group differences of each incentive factor and its influencing mechanism by ANOVA and generalized linear regression model. The innovations: from the perspective of immigrants, this study analyze the game relationship and incentive mechanism between the government and immigrants based on economic factors and behavioral perceptions. Secondly, it provides a comprehensive analytical framework for the design, configuration and balance of the government’s incentive measures. The correlation of behavior and outcome between the motivation subject government and the motivation object government is comprehensively interpreted, and its internal mechanisms and institutional obstacles are clarified. The third contribution of this study is that it broadens the scope, level and object of incentive research as well as the context structure of incentive mechanism design. The conclusions are as follows.

Non-economic incentives have been rated higher in Wuwei City than economic incentives, and there is a significant regional difference as well as a significant difference in commitment and trust between groups. The incentive effect of non-economic incentive factors is much higher than that of negative behavior evaluation, and regional differences are significant. Tianzhu County is characterized by a significant difference in cooperation intention and tendency to leave. Most of the immigrants in Wuwei City are hierarchist, egalitarian and individualist. Except for communication and opportunistic behavior, there is no difference in government incentive factors and incentive effect among cultural types. The government’s economic incentive will significantly increase the possibility of negative behaviors, while the participation incentive will effectively dispel it. The negative behaviors of immigrants are significantly affected by their cultural types. Individualists, egalitarians and autonomists are more likely to exhibit negative behaviors than fatalists. The government’s economic incentives are significantly better at promoting the incentive effect than non-economic incentives, but in dispelling the negative behavior of immigrants, non-economic incentives play a significantly higher role than economic incentives. In the future, more attention should be given to the role of participation incentives in the relocation process. Immigrants at the northern foothills of the Qilian Mountains should be encouraged to participate in specific links of decision-making, implementation, development and construction of the project. Local governments should actively listen to the needs, opinions and suggestions of migrants, improve the democratic nature of the policy, and let migrants fully appreciate the sense of gain, security, trust and significance of the relocation process. Second, non-economic incentive factors (commitment and communication incentive) should be combined with the government’s economic incentives. Oriented toward livelihood, this maximizes the positive effects of its vision, spirit, publicity and development incentive, decreases the perception of immigrants’ deprivation of power, space and development opportunities during the spatial reconstruction process, cultivating the cultural characteristics of struggle.

Designing incentive mechanisms for emigration is a comprehensive and systematic task. As this study is only based on a random survey of 131 immigrants, the characteristics of resettlement types and relocation periods of immigrants are insufficiently considered. To verify the validity of the above results, long-term dynamic tracking research with more sample groups is required. Future research can be based on the theory of planned behavior theory and social exchange to further enhance the analytical framework for incentive mechanisms. A structural equation model was applied to investigate the relationship between subjective norms of incentive mechanisms, positive and negative behavioral attitudes, perceptions of risk and incentive effects, relocation experience and departure tendency among groups that relocated for the first time, relocated for five years and relocated for ten years. Identify the direct, indirect, and moderating effects of incentive factors to choose the path, clarify the variable connection and influence mechanism, and draw the path coefficient diagram of immigrant behavioral intentions. Additionally, the mvQCA is used to analyze multiple concurrent causality between different incentives and their combinations, and a SD model is used to simulate the effectiveness and feasibility of various incentive factors and combinations under a variety of conditions, thus allowing differentiated incentive schemes to be proposed to meet the needs of different migrants.

## Figures and Tables

**Table 1:** Evaluation index system of motivation mechanism of immigration.

Target	Dimension	Indicator	Measurement Question	Mean	Weight			
					Entropy	CRITIC	CV	Final
Government motivation factor	Economic incentive	Economic incentive	The compensation and support conditions offered by the local government are very attractive to us.	3.641	0.108	0.136	0.115	0.120
	Commitment incentive	Participation motivation	The local government places a high value on our cooperative relationship and listens to our suggestions and feedback.	3.366	0.125	0.098	0.115	0.113
		Respect incentives	The local government will make some adjustments to meet our requirements.	3.366	0.078	0.084	0.096	0.086
		Vision incentive	The local government has done a lot of work for the long-term development of the migrant.	3.305	0.141	0.12	0.127	0.129
	Trust motivation	Trust motivation	The local government will see things from the immigrant’s point of view	3.420	0.162	0.108	0.131	0.133
		Development Incentive	The local government is able to give reasonable and effective suggestions for relocation, which are carefully considered.	3.542	0.067	0.082	0.090	0.080
		Spiritual motivation	The local government is concerned about our safety, particularly in matters closely related to our interests as immigrants.	3.420	0.152	0.099	0.124	0.125
	Communication incentive	Promotional incentives	The local government will inform us of the country’s various immigration policies and regulations.	3.885	0.072	0.141	0.095	0.103
		Communication motivation	The local government often discusses relocation with us.	3.527	0.094	0.132	0.107	0.111
Negative behavior of immigrants	Opportunistic behavior	Profit behavior	It is not uncommon for us to lie to protect or maximize profit when it comes to housing, land allocation and compensation.	1.878	0.480	0.254	0.378	0.370
		Default behavior	Our best interests sometimes lead us to violate formal agreements with local governments.	1.893	0.451	0.257	0.405	0.371
	Leave trend	Moving tendency	In the near future, I plan to move to other places or move back to my hometown.	3.740	0.069	0.489	0.218	0.259
The incentive effect of immigration	Response	Migration willingness	Our family is more than willing to relocate to the new village.	3.763	0.256	0.228	0.236	0.240
	Approve	Paradise recognition	In our minds, the new village represents a beautiful paradise.	3.725	0.195	0.193	0.209	0.199
		Immigration recognition	We will praise the work of the local government to others (e.g., the media, friends and family).	3.168	0.188	0.191	0.204	0.194
	Cooperation	Overall consciousness	To meet the government’s requirements, we will make some compromises and concessions.	3.878	0.191	0.178	0.143	0.171
		Advice feedback	In a timely manner, we will provide feedback and suggestions to the local government.	3.405	0.170	0.210	0.208	0.196

**Table 2:** The impact of motivation factor out of economy on negative behavior of immigrants.

Independent Variables	1 Negative Behavior		2 Negative Behavior		3 Negative Behavior		4 Negative Behavior		5 Negative Behavior		6 Negative Behavior		7 Opportunistic Behavior		8 Leave Trend	
	Beta	*t*-Test	Beta	*t*-Test	Beta	*t*-Test	Beta	*t*-Test	Beta	*t*-Test	Beta	*t*-Test	Beta	*t*-Test	Beta	*t*-Test
Constant	0.237 ***	3.105	0.321 ***	3.547	0.197 *	1.69	0.332 ***	3.327	0.297 **	2.462	0.237 *	1.937	0.034	0.268	0.204	4.205
Economic incentive	0.010	0.702	0.030 **	2.027	0.032 **	2.153	0.030 *	1.874			0.032 **	2.021	0.024	1.468	0.008	1.282
Participation motivation			−0.069 ***	−3.424	−0.067 ***	−3.331	−0.069 ***	−3.243	−0.06 ***	−2.802	−0.067 ***	−3.141	−0.056 **	−2.51	−0.012	−1.401
Respect incentives			−0.008	−0.332	−0.015	−0.638	0	0.004	−0.008	−0.29	−0.007	−0.273	−0.015	−0.55	0.008	0.753
Vision incentive			0.021	1.318	0.019	1.155	0.025	1.515	0.024	1.45	0.022	1.313	0.029 *	1.705	−0.007	−1.131
Trust motivation							−0.031	−1.379	−0.03	−1.273	−0.017	−1.042	−0.02	−0.834	−0.004	−0.463
Development Incentive							−0.016	−0.630	−0.005	−0.207	0.028	−0.664	−0.014	−0.54	−0.003	−0.273
Spiritual motivation							0.033	1.481	0.037	1.597	0.022	1.206	0.034	1.449	−0.007	−0.730
Promotional incentives					0.025	1.452			0.018	1.031	0.004	1.249	0.025	1.372	−0.003	−0.422
Communication incentive					0.007	0.428			0.003	0.151	0.019	0.210	−0.005	−0.275	0.009	1.253
Gulang County	−0.045	−1.158	−0.010	−0.250	0.004	0.100	−0.019	−0.488	−0.016	−0.403	−0.007	−0.177	0.012	0.288	−0.019	−1.198
Liangzhou District	0.028	0.641	0.012	0.286	0.013	0.310	0.011	0.247	0.023	0.503	0.010	0.230	0.068	1.462	−0.057 ***	−3.234
Junior high school	0.016	0.460	0.042	1.216	0.045	1.286	0.047	1.359	0.039	1.121	0.050	1.419	0.032	0.897	0.017	1.249
High school	−0.038	−0.779	−0.042	−0.915	−0.035	−0.752	−0.039	−0.835	−0.028	−0.593	−0.034	−0.734	−0.049	−1.014	0.015	0.789
Undergraduate	−0.027	−0.314	−0.029	−0.344	−0.036	−0.428	−0.041	−0.492	−0.043	−0.507	−0.046	−0.545	−0.082	−0.950	0.036	1.094
Individualism	0.235 ***	4.272	0.215 ***	4.065	0.219 ***	4.153	0.232 ***	4.331	0.231 ***	4.254	0.232 ***	4.339	0.22 ***	3.981	0.012	0.590
Egalitarianism	0.133 **	2.485	0.144 ***	2.829	0.166 ***	3.149	0.139 ***	2.700	0.162 ***	2.990	0.156 ***	2.922	0.146 ***	2.647	0.010	0.487
Hierarchy	0.051	1.037	0.082 *	1.734	0.095 **	1.975	0.082 *	1.731	0.082 *	1.687	0.093 *	1.921	0.055	1.100	0.038 **	1.989
Autonomy	0.309 ***	2.620	0.342 ***	2.994	0.389 ***	3.323	0.338 ***	2.954	0.340 ***	2.863	0.378 ***	3.184	0.340 ***	2.781	0.037	0.798
Tibetan	0.006	0.074	0.021	0.291	0.029	0.410	0.024	0.331	0.032	0.442	0.032	0.440	0.022	0.292	0.010	0.352
Adjusted R^2^	0.149		0.235		0.241		0.238		0.216		0.237		0.222		0.063	
Durbin−Watson	1.537		1.630		1.629		1.726		1.758		1.711		1.555		1.955	

Note: *, ** and *** represent significant at 10%, 5% and 1% confidence levels, respectively.

**Table 3:** The impact of motivation factors of economy on incentive effect of immigrants.

Independent Variables	1 Incentive Effect		2 Incentive Effect		3 Incentive Effect		4 Incentive Effect		5 Incentive Effect		6 Incentive Effect		7 Behavioral Response		8 Effect of Recognition		9 Cooperation Intention	
	Beta	*t*-Test	Beta	*t*-Test	Beta	*t*-Test	Beta	*t*-Test	Beta	*t*-Test	Beta	*t*-Test	Beta	*t*-Test	Beta	*t*-Test	Beta	*t*-Test
Constant	0.512	8.942	0.381 ***	5.488	0.398 ***	4.562	0.414 ***	4.668	0.317 ***	3.517	0.267 ***	2.926	0.016	0.338	0.068	1.475	0.182 ***	3.573
Economic incentive	0.046 ***	4.213	0.036 ***	0.002	0.048 ***	4.371					0.027 **	2.298	0.024 ***	3.723	0.008	1.265	−0.004	−0.606
Participation motivation			0	−0.021			0.016	0.996	−0.008	−0.486	−0.014	−0.877	−0.001	−0.159	−0.005	−0.575	−0.008	−0.885
Respect incentives			0.025	1.371			0.022	1.165	0.006	0.297	0.006	0.327	−0.003	−0.283	−0.016	−1.592	0.025 **	2.321
Vision incentive			0.025 **	2.081			0.027 **	2.104	0.019	1.539	0.017	1.390	−0.003	−0.490	0.014 **	2.294	0.006	0.857
Trust motivation									0.001	0.074	0.006	0.348	0.008	0.804	0.015 *	1.710	−0.017 *	−1.707
Development Incentive									0.045 **	2.400	0.035 *	1.858	0.006	0.620	0.013	1.349	0.016	1.506
Spiritual motivation									0.029 *	1.668	0.021	1.232	0.020 **	2.230	0.006	0.722	−0.006	−0.592
Promotional incentives					0.027 **	2.010	0.014	0.990	0.018	1.357	0.021	1.613	0.005	0.770	0.006	0.855	0.010	1.366
Communication incentive					−0.003	−0.259	−0.001	−0.041	−0.011	−0.836	−0.010	−0.786	−0.013 *	−1.844	0.002	0.275	0.001	0.111
Gulang County	−0.090 ***	−3.122	−0.086 ***	−2.951	−0.078 ***	−2.662	−0.090 ***	−2.908	−0.085 ***	−2.852	−0.077 ***	−2.627	−0.032 **	−2.04	−0.009	−0.584	−0.037 **	−2.221
Liangzhou District	−0.087 ***	−2.611	−0.063 *	−1.877	−0.084 **	−2.554	−0.056	−1.612	−0.029	−0.850	−0.039	−1.171	0.016	0.918	−0.002	−0.122	−0.053 ***	−2.858
Junior high school	0.047 *	1.791	0.024	0.923	0.049 *	1.875	0.016	0.573	0.014	0.526	0.023	0.865	0.006	0.440	0.011	0.861	0.005	0.331
High school	0.062 *	1.681	0.060 *	1.683	0.066 *	1.806	0.070 *	1.891	0.066 *	1.852	0.060 *	1.730	0.006	0.333	0.011	0.648	0.042 **	2.185
Undergraduate	0.016	0.246	−0.006	−0.100	0.001	0.023	−0.008	−0.123	−0.010	−0.159	−0.012	−0.197	0.001	0.036	0.020	0.625	−0.033	−0.941
Individualism	−0.013	−0.308	−0.002	−0.053	−0.008	−0.198	−0.005	−0.107	−0.002	−0.055	−0.001	−0.025	0.011	0.500	−0.018	−0.897	0.007	0.296
Egalitarianism	−0.073 *	−1.814	−0.067 *	−1.701	−0.057	−1.384	−0.052	−1.241	−0.035	−0.875	−0.040	−1.009	−0.014	−0.677	−0.002	−0.088	−0.024	−1.077
Hierarchy	0.022	0.590	0.015	0.417	0.033	0.892	0.008	0.198	0.022	0.597	0.031	0.856	0.019	0.995	0.021	1.150	−0.009	−0.474
Autonomy	−0.134	−1.513	−0.113	−1.297	−0.094	−1.041	−0.133	−1.425	−0.117	−1.323	−0.086	−0.971	−0.053	−1.132	−0.089 **	−1.977	0.055	1.124
Tibetan	0.029	0.517	0.039	0.724	0.041	0.736	0.054	0.939	0.029	0.537	0.029	0.537	−0.019	−0.672	0.013	0.492	0.034	1.147
Adjusted R^2^	0.246		0.291		0.259		0.229		0.309		0.335		0.293		0.283		0.171	
Durbin-Watson	2.027		1.969		1.975		1.990		2.078		2.025		2.173		1.645		2.100	

Note: *, ** and *** represent significant at 10%, 5% and 1% confidence levels, respectively.

## Data Availability

The data presented in this study are available on requested from the author.
